# Evaluation of the Complex Nomenclature of the Clinically and Veterinary Significant Pathogen* Salmonella*

**DOI:** 10.1155/2017/3782182

**Published:** 2017-04-30

**Authors:** Michael P. Ryan, Jean O'Dwyer, Catherine C. Adley

**Affiliations:** ^1^Industrial Biochemistry Programme, Department of Chemical Sciences, School of Natural Sciences, University of Limerick, Limerick V94 T9PX, Ireland; ^2^Department of Biological Sciences, School of Natural Sciences, University of Limerick, Limerick V94 T9PX, Ireland; ^3^Microbiology Lab, School of Natural Sciences, University of Limerick, Limerick V94 T9PX, Ireland

## Abstract

*Salmonella* encompasses a vast and highly related population of clinically and veterinary significant pathogens. The genus is responsible for an array of diseases such as typhoid fever and salmonellosis (a variety of illnesses including gastroenteritis), which cause public health issues globally. Even with the global recognition of* Salmonella* as a significant human and veterinary pathogen, the highly complex and evolving nomenclature system of* Salmonella* is problematic for clinicians, veterinarians, and microbiologists to comprehend. The present paper offers a review of the ever developing nomenclature for this bacterial species.

## 1. Introduction

*Salmonella* is a genus in the family Enterobacteriaceae which are Gram-negative, oxidase negative, catalase positive, nonspore forming rods. They are also facultative anaerobes. Almost all* Salmonella* species are motile via peritrichous flagella, with the poultry pathogen* Salmonella enterica* ser. Gallinarium being a noteworthy exception [1, 2]. In terms of distribution,* Salmonella* are extensively represented within the environment and can cause a wide range of illnesses in both human and animals. In humans, infection with* Salmonella* can cause several different illnesses such as typhoid fever, septicaemia, localized infections of various bodily tissues, and gastroenteritis [3]. Nontyphoidal* Salmonella* spp. alone were estimated to have caused 1,027,561 illnesses (and 378 deaths) in the US in 2011 [4].

Salmonellae optimal growth temperature is 37°C; however growth has been recorded between 2 and 4°C and as high as 54°C [4].* Salmonella* can live in a wide pH range from as low as pH 3.8 to as high as pH 9.5 with an optimum of pH 6.5–7.5 [5]. A water activity (*a*_*w*_) of less than 0.94 is inhibitory to* Salmonella* growth [6]; however, at certain temperatures a low *a*_*w*_ is believed to have a protective effect on* Salmonella* [7, 8]. Biochemical features used to identify* Salmonella* include hydrogen sulphide production, lysine and ornithine decarboxylation, and nonhydrolysation of urea [5].

In the past, the classification of* Salmonella* strains was founded on a mixture epidemiology: isolate host range, the clinical expression of infection, biochemical reactions, and the antigenic pattern of the isolate [9].

Since its first isolation, several different nomenclatural systems have been used for these bacteria which split the genus into various different subgenera, species, subspecies, subgenera, groups, subgroups, and serovars [10] in an inconsistent manner which we will elucidate within this review paper.

## 2. *Salmonella* Taxonomy and Nomenclature

*Salmonella* was given its name after Daniel E. Salmon who was the veterinary surgeon that first isolated (what was called at the time)* “Bacillus choleraesuis”* from porcine intestines in 1884 [11, 12]. This name was changed in 1900 to “*Salmonella choleraesuis*” by Lignieres [13].

Today the* Salmonella* genus is split into just 2 species:* Salmonella enterica* and* Salmonella bongori*, with* S. enterica* being split into 6 additional subspecies. In the past* S*.* enterica* subspecies were thought to be subgenera and serovars/serotypes of* Salmonella* were considered to be separate species, which, if still followed today, would result in greater than 2600 species of* Salmonella* [14]. The terms “serovars” and “serotypes” are generally considered to be synonymous. The World Health Organisation (WHO)/Institut Pasteur use the term “serovar,” while the Centres for Disease Control (CDC) and the American Society of Microbiology (ASM) originally used the word “serotype” but have steadily changed it to “serovar” in order to maintain international consistency. In this the paper the term “serovar” is used.

## 3. Multiple Species?

In 1966 it was proposed by Kauffmann that every* Salmonella* serovar is thought of as individual separate species [15]. All serovars (before and after 1966) were initially designated by antigenic formula. Prior to 1966, serovar “names” were assigned irrespective of subspecies, for example,* Salmonella* Marina (subsp. IV 48:g,z51:-),* Salmonella* Bongor (subsp. V 48:z35:-), and* Salmonella* Daressalaam (subsp. II 9,12:l,w:e,n,x). Serovars were named owning the disease (*S*. Typhi) and/or the animal (*S*. Typhimurium) the bacterium had been isolated from or the geographic location the serovar had originally been isolated from, for example,* S*. Kentucky and *S*. Dublin. After 1966, names for nonsubspecies I Salmonellae were withdrawn from the scheme. These serovars are now referred to by antigenic formula alone (this will be discussed further below). It however took practitioners many years to start following these guidelines leading to more confusion.

## 4. Classification into Subspecies

Due to the confusion surrounding the use of multiple* Salmonella* species, Borman et al. suggested that the* Salmonella* genus be split into three species,* Salmonella choleraesuis,* which would be the type species of the* Salmonella* genus, “*Salmonella typhosa*” (*S.* Typhi renamed), and “*Salmonella kauffmannii*” with “*S. kauffmannii*” encompassing every other* Salmonella* serovar [16]. This recommendation was however ignored. Later, “*Salmonella enterica*” was proposed to encompass all Salmonellae (Kauffmann and Edwards, 1952) [17]. Following this, a comparable three-species naming system to that of Borman et al. was suggested in 1966. In this system, the name “*Salmonella enteritidis*” would signify all serovars other than “*Salmonella typhosa”* and* Salmonella choleraesuis* [18]. In 1970, Le Minor et al. laid down recommendations that the “subgenera” laid out by Kauffmann and Edwards be considered species. This led to names “*Salmonella kauffmannii*” beginning designated for “subgenus” I,* Salmonella salamae* for “subgenus” II,* Salmonella arizonae* for “subgenus” III, and* Salmonella houtenae* for “subgenus” IV [19]. Serovars of “*S. kauffmannii*” would be named by their species names followed by that of their serovar (for example, “*S. kauffmannii*” serovar Paratyphi). Serovars of the other “subgenus” listed above would be named by their species names succeeded by their antigenic make up.

## 5. One Species?

In the early 1970s, using DNA-DNA hybridisation techniques, Crosa et al. stated that the* Salmonella* species and serovars of* Arizona* were highly related to one another and should thus be designated as the one species [20, 21]. Le Minor et al. put forward a proposal that there were a single* Salmonella* species and seven subspecies based on DNA relatedness studies. The name “*Salmonella choleraesuis*” was chosen [22]. Under the changes to the nomenclature system proposed by these authors the name of a* Salmonella* serovar should not be italicised or underlined, for example,* Salmonella choleraesuis* subsp.* choleraesuis* ser. Choleraesuis/Typhi.

## 6. “*Salmonella choleraesuis*” as the Type Species?

Due to the problems surrounding the use of “choleraesuis” as a designation (denoting a species as well as a serovar led to confusion) it was suggested again in 1986 that “*Salmonella enterica*” be used as the designation of the* Salmonella* type species by the Subcommittee of Enterobacteriaceae of the International Committee on Systematic Bacteriology at the XIV International Congress of Microbiology [23].

Le Minor and Popoff (World Health Organisation) formally proposed to the Judicial Commission of the International Committee of Systematic Bacteriology that* S. enterica* be the type and only species of* Salmonella* in 1987. The name “enterica” was recommended for use as this had not been used as a name for a serovar. The proposal laid out a nomenclature scheme where* Salmonella* was divided into 7 subspecies* Salmonella enterica* subsp.* enterica* (I),* Salmonella enterica* subsp.* salamae* (II),* Salmonella enterica* subsp.* arizonae* (III),* Salmonella enterica* subsp.* houtenae* (IV),* Salmonella enterica* subsp.* bongori* (V), and* Salmonella enterica* subsp.* indica* (VI) [24]. Subspecies III was split into subspecies* Salmonella enterica* subsp.* arizonae* (IIIa) and* Salmonella enterica* subsp.* diarizonae* (IIIb), based upon DNA comparisons and biochemical utilisation patterns. One* Salmonella* subspecies, Sa*lmonella enterica* subsp.* bongori* (subspecies V), was given separate species status. This decision was due to differences between this species and the other* Salmonella* subspecies seen in DNA relatedness studies and multilocus enzyme electrophoresis techniques [25].

These proposals were rejected by the committee over fears that* Salmonella* Typhi may be overlooked if called* Salmonella enterica* subsp.* enterica* serovar Typhi. The system however was adopted by a number of organisations across different countries including the ASM, WHO, and CDC [9]. However* S. choleraesuis* was kept as the type species awaiting a request for opinions on the matter [26].

Several requests for opinions were published some in agreement and others against the 1987 proposals of Le Minor and Popoff. In particular, Euzéby called for the use of* S*.* enterica* as the type species but maintaining* S.* Typhi as a species [27]. Ezaki et al. [28] suggested the upgrading of* Salmonella choleraesuis* subsp.* choleraesuis* serovar Paratyphi to full species level. An additional recommendation by Ezaki et al. [29] further called for the preservation of* Salmonella enteritidis* and* Salmonella typhimurium* at the species level. Yabuuchi and Ezaki requested the maintenance of* Salmonella choleraesuis* as the type species and a change in name of* Salmonella choleraesuis* subsp.* choleraesuis* serovar Choleraesuis to* Salmonella choleraesuis* subsp.* choleraesuis* serovar Hogcholera [30].

In 2002, the Judicial Commission of the International Committee for Systematics of Prokaryotes in the Judicial Opinion 80 carefully discussed the request for classification of* Salmonella* nomenclature. They approved the request for the change in the nomenclature system for* Salmonella* and from January 2005, “*Salmonella choleraesuis*” would change to “*Salmonella enterica*” with “*Salmonella enterica*” becoming the type species of the genus* Salmonella*. The commission decision conforms to the bacteriological code; however it falls short of abolishing the use of* S. choleraesuis* as the type strain [31]. The requests by Euzéby [27], Ezaki et al., [28, 29], and Yabuuchi and Ezaki [30] were not accepted [32].

The Judicial Commission ruled that the* Salmonella* genus is comprised of two species, called “*Salmonella enterica*” and “*Salmonella bongori*.” “*Salmonella enterica*” comprises six subspecies, “*Salmonella enterica* subsp.* enterica*,” “*Salmonella enterica* subsp.* salamae*,” “*Salmonella enterica* subsp*. arizonae*,” “*Salmonella enterica* subsp.* diarizonae*,” “*Salmonella enterica* subsp.* houtenae*,” and “*Salmonella enterica* subsp.* indica*.” An accompanying commentary was written to help better understand the consequences to both the nomenclature and taxonomy of* Salmonella* due to Opinion 80 [33].

## 7. *Salmonella* Serovars

Serotyping is a serological procedure which separates strains of microorganisms into different groups based on their antigenic composition. Conventional serotyping or antigenic classification of* Salmonella* was traditionally founded upon antibody reaction with 3 types of surface antigens: somatic O antigens, flagellar H antigens, and Vi capsular antigens (Nataro et al., 2011) [34]. The O antigen determines the group the* Salmonella* isolate belongs to while the H antigen determines the serovar [35]. Serotyping may now be extrapolated by characterization of O and H antigen genes. The capsular antigen occurs only in* S.* Typhi,* S.* Paratyphi C (this antigen is not found in Paratyphi A or B), and* S.* Dublin [5].

The O antigen is a heat stable polysaccharide present on the outer surface of the lipopolysaccharide. Each O antigen is composed of 5-6 sugar units, variation in the sugar units, covalent bonds between sugar units, and linkage between O antigen subunits, resulting in different O antigens [5]. O antigen identification is carried out in two parts. Firstly, the isolate is tested using O grouping sera by slide agglutination. Once the O group is identified tests are then carried out with specific antisera that react with individual antigens [34].

The Vi capsular antigen is most commonly found in* S.* Typhi but is also occasionally identified in* S.* Dublin,* S.* Paratyphi C, and some* Citrobacter* strains. Slide agglutination with specific antiserum is used to identify the Vi capsular antigen [34].

H antigens are composed of flagellin subunits and are the filamentous portion of the bacterial flagella [34].* Salmonella* serovars express either one type of H antigen, that is, monophasic, or two types of H antigen, that is, diphasic.* Salmonella* is unique amongst enteric bacteria in this regard. Both phases may be detected in a culture as a whole but it is believed individual cells of diphasic isolates only express H antigen from one phase at a time. Tube agglutination or slide agglutination tests are performed to determine the H antigen.* Salmonella* isolates are tested firstly with H typing antisera which recognise multiple antigens and then with H single factor antisera which identify specific antigens [34].

## 8. Current Nomenclature System Used by the WHO and ASM

### 8.1. Species Name

The nomenclature system outlined by Tindall et al. is the system that is currently used by the WHO, and the ASM [33].

### 8.2. Serovars

It is important to note that the term “serovar” can have two meanings in the context of* Salmonella* with “serovar” meaning both the antigenic formula (discussed below) of the various subspecies and the serovar name which is used in the practice of assigning formal names to isolates of* S. enterica* subsp.* enterica* (subspecies I). For* Salmonella enterica* subsp.* enterica*, historically the names of the serovars were chosen due to diseases associated with infection, the geographic area of their isolation, or typical habitats. Currently however serovars names are assigned solely based upon geographic place names related to the area where the serovar was first isolated. In the other* Salmonella enterica* subspecies and in* S. bongori* serovars, antigenic formulae are assigned using the Kauffmann-White-Le Minor scheme [36]. For the first mention in a publication, the full name “*Salmonella enterica”* is used. Following* Salmonella enterica,* the subspecies is named (*Salmonella enterica* subsp.* enterica*). This is then followed by the word “serovar” or the abbreviated version “ser.” along with the name of the serovar. The name of the serovar is given in nonitalicised Roman alphabet letters with the first letter capitalised. Therefore the full name would be, for example,* Salmonella enterica* subsp./ssp.* enterica* ser. Typhi. Subsequent mentions of the name can be condensed with “*Salmonella*” being followed by just the serovar name, for example,* Salmonella* Typhi or* S*. Typhi [37]. Serovars of* S. enterica* subspecies* enterica* are normally named after the location they were first identified [38]. A full serovar name is only assigned to isolates of* Salmonella enterica* subsp.* enterica* which meet the full antigenic definition for a serovar. For example,* Salmonella enterica* subsp.* enterica* 4,5,12:i:- is a commonly encountered, monophasic variant of* Salmonella enterica* subsp.* enterica* ser. Typhimurium. These strains fail to express the fljB-encoded phase-2 flagellar antigen [39]. As they lack a full antigenic formula they cannot be given a full name. For strains such as this and other antigenic variants, the antigenic formula becomes the serovar name.

For the other five* Salmonella enterica* subspecies serovars are designated according to antigenic formulae, the subspecies name is given in Roman letters (not italicised), and antigenic formulae are then listed as follows: O (somatic) antigens: Vi (when present): H (flagellar) antigens (phase 1): H antigens (phase 2, if present). A colon is used to separate each antigen, for example,* Salmonella* subsp. II 58: l,z_13_,z_28_: z6 [34]. For serovars of* S. bongori* (which was formerly classed as subgenus V), V is still used in order to be consistent with the scheme used for* Salmonella enterica*, for example,* S*. V 48:z35:- [38].

When first published in 1934 the Kauffmann-White scheme included 44 serovars. Following Kauffmann's retirement in 1964 there were 958 serovars listed. When Le Minor retired, the list contained 2267 serovars and following Popoff's retirement there were 2555 serovars. To acknowledge the work done by Le Minor, the leaders of the committee in charge of the scheme, Grimont and Weill, proposed to change the name of the Kauffmann-White scheme to White-Kauffmann-Le Minor scheme [34].

**Figure 1:**
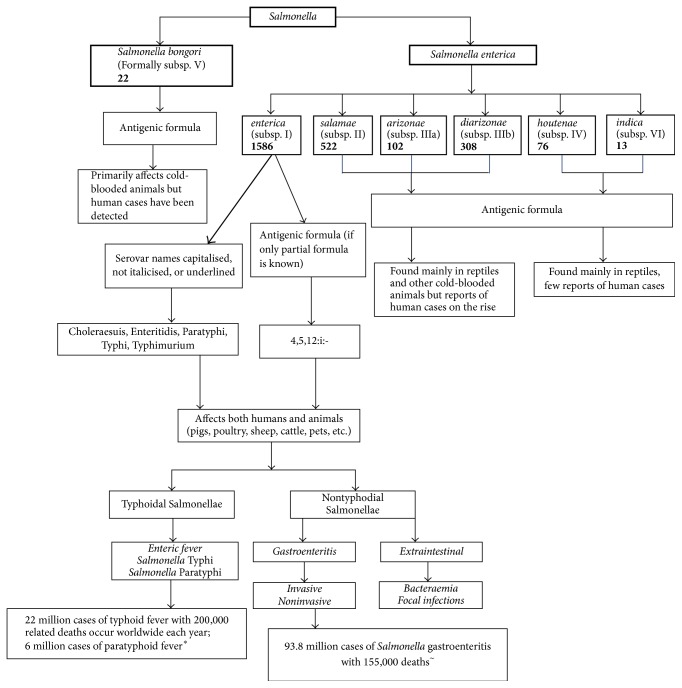
*Salmonella* genus nomenclature breakdown. ^*∗*^[40], ^~^[41].

The present classification system is founded upon the antigenic determinants of various* Salmonella* serovars and the system in use today has been built up over 80 years of research on antibody interactions with the surface antigens of* Salmonella* bacterium. The antigenic formulae of all known* Salmonella* serovars are recorded in the Kauffmann-White-Le Minor scheme [36]. The World Health Organisation Collaborating Centre for Reference and Research on* Salmonella* is located at the Pasteur Institute in Paris which maintains and updates the scheme. Newly identified serovars are published in the journal* Research in Microbiology* by those in control of the scheme. In the last update published in 2014 (an update to 2010), there were 2659 serovars in the genus* Salmonella* (2639 in* Salmonella enterica* and 20 in* Salmonella bongori*). A full breakdown of the numbers belonging to each subspecies can be seen in Figure 1 [14].

## 9. Conclusion

*Salmonella* spp. are still one of the most infectious food borne pathogens causing considerable issues worldwide in both human and veterinary medicine. Even with this global recognition of* Salmonella* as a significant human and veterinary pathogen, its highly complex nomenclature system is problematic for clinicians, veterinarians, and microbiologists to comprehend. This paper addresses the complexity for practitioners use.
